# Parameters and computer software for the evaluation of mass attenuation and mass energy-absorption coefficients for body tissues and substitutes

**DOI:** 10.4103/0971-6203.35725

**Published:** 2007

**Authors:** Akintunde A. Okunade

**Affiliations:** Department of Physics, Obafemi Awolowo University 220005, ILE-IFE Osun State, Nigeria

**Keywords:** Body tissues and substitutes, mass attenuation coefficient, mass energy-absorption coefficient

## Abstract

The mass attenuation and energy-absorption coefficients (radiation interaction data), which are widely used in the shielding and dosimetry of X-rays used for medical diagnostic and orthovoltage therapeutic procedures, are strongly dependent on the energy of photons, elements and percentage by weight of elements in body tissues and substitutes. Significant disparities exist in the values of percentage by weight of elements reported in literature for body tissues and substitutes for individuals of different ages, genders and states of health. Often, interested parties are in need of these radiation interaction data for body tissues or substitutes with percentage by weight of elements and intermediate energies that are not tabulated in literature. To provide for the use of more precise values of these radiation interaction data, parameters and computer programs, MUA_T and MUEN_T are presented for the computation of mass attenuation and energy-absorption coefficients for body tissues and substitutes of arbitrary percentage-by-weight elemental composition and photon energy ranging between 1 keV (or k-edge) and 400 keV. Results are presented, which show that the values of mass attenuation and energy-absorption coefficients obtained from computer programs are in good agreement with those reported in literature.

In the applications of X-ray photons for medical diagnostic and therapeutic purposes, the quantity of X-ray photons that are absorbed and transmitted after interaction within the human tissue and materials of biological interest can be theoretically evaluated by using linear (or mass) attenuation coefficients. The theoretical evaluation of the absorbed dose to human tissue from X-ray photons can be carried out using mass energy-absorption coefficients.[1–6] Both the types of coefficients, which are widely used in shielding and dosimetric computation, are strongly dependent on the energy of photon, elements and the percentage by weight of the elements in the medium within which the photon interacts. As a result of the tremendous usefulness of these coefficients in the modeling of the transport and dosimetry of photons in biological and shielding materials, works resulting in extensive database over a period of decades have been published.[7–16] The parameterization of these interaction data has been reported to promote the ease of use in theoretical simulations of transport and dosimetry of photons in medical and biological applications. Several works on the parameterization of mass attenuation and mass energy-absorption coefficients have been reported in literatures.[17–33] Parameterization studies such as those reported by some workers[17,18,21,22,27,29,31–33] are considered complex since they are based on physical quantities such as electron density and cross section per electron. Simple polynomial functions were used in other parameterization schemes reported for the evaluation of mass attenuation and energy-absorption coefficients.[19,20,23,24,28,30] Some of these schemes were obtained by using older interaction data[9,10,11,14] and do not cover the whole diagnostic and orthovoltage energy range. Some workers[12,13] developed a computer program, XCOM, in FORTRAN language for the calculation of mass attenuation coefficients for any element, compound and mixture at energies ranging from 1 to 100 GeV. The computation of mass energy-absorption coefficients was not addressed in XCOM. The Windows version of XCOM called WinXCom has been reported.[34] Also, a computer program with acronym XMuDat[35] has been published for the computation of mass attenuation and mass energy-absorption coefficients for 290 elements, compounds and mixtures. This program limits the choice of the constituents (element, compound or mixture) in the absorber material to a maximum of six.

Specifically for optimal assessments of the use of X-rays for medical purposes, accurate and appropriate X-ray photon interaction coefficients of various normal and diseased body tissues are required. In theoretical simulation exercises, researchers are often in need of radiation-interaction data for tissues or ‘tissue substitutes’ of elemental composition and weight percentages which differ from those reported in literature. More than six constituents are required for some of these tissues and substitutes. To meet the need for photon interaction data at low-energy photons (1 keV or k-edge – 400 keV) for body tissues or substitutes of interest and arbitrary percentage-by-mass weighting of elemental composition, segmented multifits to the mathematical expression reported[23] for mass attenuation and mass energy-absorption coefficients of major and some trace elements in body tissues are presented.

## Materials and Methods

The least-square curve fits to values of mass attenuation and mass energy-absorption coefficients were carried out using more recent data.[13] These interaction data are based on more recent calculations[36] and replace those earlier reported.[11] Fitted parameters were obtained for equations of the forms:[23]

$$\text{F}\left(x\right)=a_{1}+a_{2}x^{-1.6}+a_{3}x^{-2.7}+a_{4}x^{-3.5}+a_{5}x^{-4.5}\;\mathrm{(1)}$$

where F(x)=μρ(x) or μenρ(x), ai'S (in units of cm^2^g^−1^) are parameters resulting in best fits, x = E/100keV and E is the X-ray photon energy in keV

Data points between 1 (or k-edge) and 400 keV were used. This energy range was divided into regions that result in good agreement with fitted data for 17 elements. These were hydrogen, carbon, oxygen, nitrogen, fluorine, sodium, magnesium, aluminum, silicon, phosphorus, sulfur, chlorine, argon, potassium, calcium, calcium and iron. For the purpose of comparison, computations were carried out for mass attenutation, $\begin{array}{c} \text{μ}\\ \text{ρ} \end{array}\left(\text{x}\right)$, and mass energy-absorption coefficients, $\begin{array}{c} {\text{μ}}_{\text{en}}\\ \text{ρ} \end{array}\left(\text{x}\right)$, for some selected materials of biological interest using mixture rule:

$$\frac{\mu }{\rho }\left(x\right)=\sum_{\text{i}}{\text{W}}_{\text{i}}{\left[\frac{\mu }{\rho }\left(x\right)\right]}_{\text{i}}\;\mathrm{(2)}$$

$$\text{and }\frac{{\text{μ}}_{\text{en}}}{\text{ρ}}\left(\text{x}\right)\text{=}\sum_{\text{i}}{\text{W}}_{\text{i}}{\left[\frac{{\text{μ}}_{\text{en}}}{\text{ρ}}\left(\text{x}\right)\right]}_{\text{i}}\;\mathrm{(3)}$$

Where ${\left[\frac{\mu }{\rho }\left(x\right)\right]}_{\text{i}}$ and ${\left[\frac{{\text{μ}}_{\text{en}}}{\text{ρ}}\left(x\right)\right]}_{\text{i}}$ are respectively attenutation, and mass energy-absorption coefficients for the *i*^th^ element in the material and w_i_ is the fraction by weight of the *i*^th^ element.

This rule is considered valid for photons in the energy range under consideration (1-400 keV). For this energy range the values of the factor ‘g,’ which represents the average fraction of the kinetic energy of secondary charged particles, are relatively small.[5] Using the results of the multifits obtained from Eq. (1), computer programs MUA_T and MUEN_T were developed (using FORTRAN language) for the computation of mass attenuation and mass energy-absorption coefficients for body tissues and substitutes of arbitrary percentage-by-mass weighting of elemental composition. These computer programs are available for download via http://okunade.phpnet.us or on request vial email from the author.

## Results

**Figure 1:**
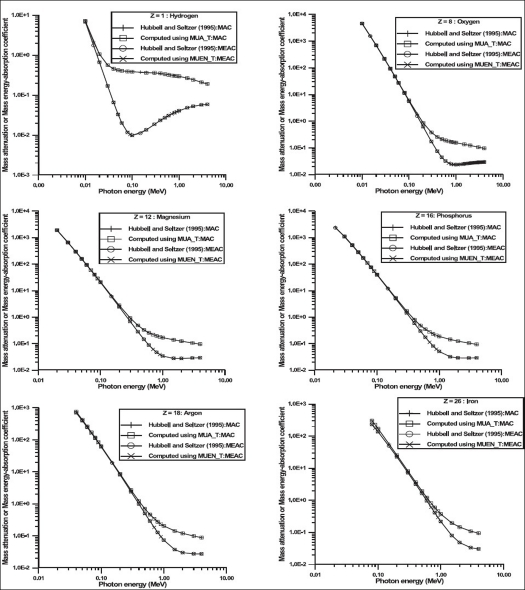
Comparison of the values of mass attenuation and mass energy-absorption coefficients obtained from Eq. 1 and fitted values for some selected elements. The acronyms MAC and MEAC stand for mass attenuation coefficient and mass energy-absorption coefficient respectively

**Figure 2:**
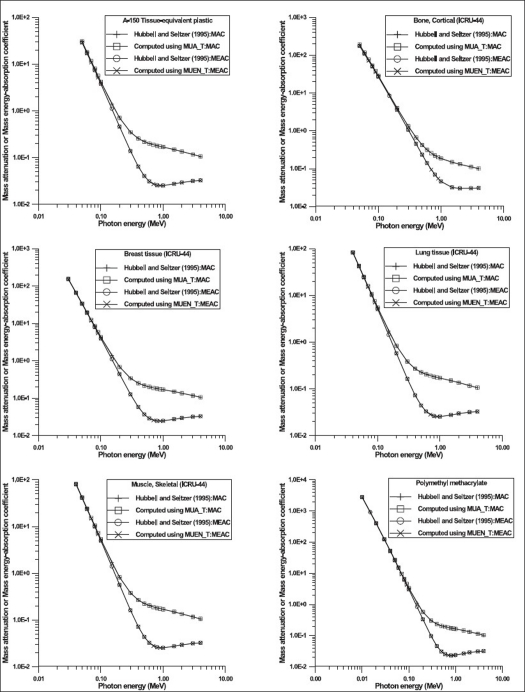
Comparison of the values of mass attenuation and mass energy-absorption coefficients obtained from Eqs. 1–3 and values from literature for some selected body tissues. The acronyms MAC and MEAC stand for mass attenuation coefficient and mass energy-absorption coefficient respectively.

**Figure 3:**
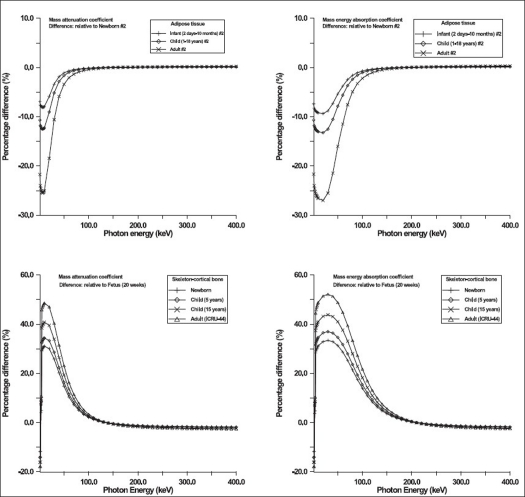
Variations in the values of mass attenuation and mass energy-absorption coefficients for some body tissues as a result of differences in age (or elemental compositions)

The values of parameters resulting in best fits to Eq. (1) are shown in Tables 1 and 2. Typical results of comparison of values of mass attenuation and mass energy-absorption coefficients obtained from Eq. (1) and fitted values are shown on Figure 1. The results of comparison of values obtained for some selected body tissues by using Eqs. 1–3 and those tabulated in literatures are shown in Figure 2. The variations in values of mass attenuation and mass energy-absorption coefficients for some body tissues as a result of differences in age are shown in Figure 3. Table 3 shows the values of elemental compositions for these selected body tissues.[37]

**Table 1:** Parameters resulting in best fit to the values of mass attenuation coefficient (from reference. 15) using Eq. 1.

*Material*	*Energy range (keV)*	*a_1_*	*a_2_*	*a_3_*	*a_4_*	*a_5_*	*Absolute maximum percentage*
Hydrogen	1-10	3.784E-1	1.659E-4	−2.847E-6	8.626E-7	−1.335E-9	<1.0
	10-80	2.357E-1	7.064E-2	−2.606E-2	5.358E-3	−2.071E-4	~1.0
	80-400	1.487E-1	5.271E-1	−7.735E-1	4.668E-1	−7.368E-2	~1.0
Carbon	1-8	1.541	−6.622E-2	6.753E-3	1.069E-4	−4.513E-7	1.7
	8-50	1.396E-1	1.624E-2	−2.479E-3	1.125E-3	−2.303E-5	1.2
	50-400	7.479E-2	2.680E-1	−3.914E-1	2.381E-1	−3.760E-2	~1.0
Nitrogen	1-8	2.370	−1.107E-1	1.186E-2	1.166E-4	−6.612E-7	1.5
	8-50	1.399E-1	1.621E-2	−1.488E-3	1.518E-3	−3.033E-5	1.1
	50-400	7.486E-2	2.674E-1	−3.873E-1	2.355E-1	−3.692E-2	~1.0
Oxygen	1-8	3.270	−1.663E-1	1.901E-2	9.118E-5	−8.367E-7	<1.0
	8-50	1.409E-1	1.532E-2	2.983E-4	1.997E-3	−3.988E-5	1.0
	50-400	7.465E-2	2.704E-1	−3.927E-1	2.412E-1	−3.795E-2	~1.0
Florine	1-8	3.868E+0	−2.111 E-1	2.682E-2	5.134E-5	−1.135E-6	1.0
	8-80	1.346E-1	1.300E-2	2.903E-3	2.409E-3	−4.800E-5	~1.3
	80-400	7.093E-2	2.512E-1	−3.486E-1	2.000E-1	−2.341E-2	1.3
Sodium	2-6	−3.685E+1	8.665E-1	−4.948E-3	2.238E-3	−1.541E-5	2.1
	6-50	1.546E-1	−5.380E-3	1.599E-2	2.933E-3	−5.286E-5	2.2
	50-400	7.169E-2	2.573E-1	−3.645E-1	2.301E-1	−3.566E-2	~1.0
Magnesium	2-8	1.241E+0	−1.976E-1	5.320E-2	3.756E-4	−8.017E-6	<1.0
	8-20	1.968E-1	−3.601E-2	3.076E-2	2.612E-3	−4.358E-5	<1.0
	20-100	1.189e-1	5.002E-2	−1.454E-2	1.519E-2	−8.127E-4	<1.0
	100-400	7.406E-2	2.630E-1	−3.569E-1	2.137E-1	−2.465E-2	~1.0
Aluminum	2-6	−9.765E+0	3.800E-1	2.494E-2	2.709E-3	−2.907E-5	~1.1
	6-50	2.091E-1	−3.979E-2	3.716E-2	3.545E-3	−7.076E-5	<1.3
	50-400	7.559E-2	2.181E-1	−2.563E-1	1.542E-1	−1.973E-2	~1.8
Silicon	2-10	5.015E+0	−5.104E-1	1.059E-1	−1.218E-3	2.097E-7	~0.5
	10-50	1.564E-1	−3.209E-4	2.933E-2	8.658E-3	−2.650E-4	~0.2
	50-400	7.812E-2	2.269E-1	−2.633E-1	1.641E-1	−2.088E-2	~1.8
Phosphorus	3-8	−1.293E+1	5.832E-1	3.026E-2	5.000E-3	−6.861E-5	<1.0
	8-50	1.631E-1	−2.468E-2	4.943E-2	7.051E-3	−1.865E-4	1.3
	50-400	7.262E-2	2.621E-1	−3.562E-1	2.458E-1	−3.718E-2	~1.0
Sulphur	3-10	−3.778E-1	2.474	−1.204E-1	1.759E-2	−2.111 E-4	1.6
	10-50	1.504E-1	−7.377E-3	5.109E-2	1.160E-2	−3.805E-4	<1.0
	50-400	7.458E-2	2.734E-1	−3.679E-1	2.621E-1	−3.947E-2	~1.0
Chlorine	3-5	1.954E+2	5.599	−8.719E-1	8.747E-2	−1.077E-3	<1.0
	5-20	6.212E-1	−2.017E-1	1.201E-1	2.194E-3	−7.789E-5	<1.0
	20-100	1.194E-1	3.718E-2	2.449E-2	2.541E-2	−1.332E-3	<1.0
	100-400	7.203E-2	2.576E-1	−3.264E-1	2.277E-1	−2.541E-2	~1.0
Argon	4-20	1.163E+0	−3.375E-1	1.582E-1	−9.313E-4	−2.768E-5	<0.1
	20-80	1.459E-1	−2.696E-2	7.743E-2	1.248E-2	−4.487E-4	<1.0
	80-400	6.772E-2	2.421E-1	−2.996E-1	2.188E-1	−2.421E-2	~1.0
Potassium	4-20	6.602E-1	−3.061E-1	1.888E-1	−8.278E-4	−4.150E-5	<1.0
	20-100	1.230E-1	3.138E-2	4.962E-2	3.260E-2	−1.820E-3	0.1
	100-400	7.315E-2	2.606E-1	−3.134E-1	2.411E-1	−2.642E-2	~1.0
Calcium	5-20	1.404E+0	−5.219E-1	2.587E-1	−5.607E-3	3.131E-5	<1.0
	20-100	1.257E-1	3.224E-2	6.292E-2	3.878E-2	−2.276E-3	0.1
	100-400	7.524E-2	2.672E-1	−3.122E-1	2.554E-1	−2.799E-2	~1.0
Manganese	8-50	4.710E-1	−3.467E-1	3.617E-1	−3.884E-3	−1.356E-4	~0.5
	50-80	9.584E-2	7.446E-2	8.275E-2	9.121E-2	−7.850E-3	~0.6
	80-400	6.899E-2	2.429E-1	−2.184E-1	2.732E-1	−2.934E-2	~1.1
Iron	8-40	5.323E-1	−3.768E-1	4.374E-1	−3.130E-3	−2.809E-4	0.1
	40-100	1.309E-1	−1.961E-2	2.120E-1	5.250E-2	−3.734E-3	0.1
	100-400	7.021E-2	2.531E-1	−2.263E-1	3.214E-1	−4.622E-2	~1.0

**Table 2:** Parameters resulting into best fit to values of mass energy-absorption coefficients (from reference. 15) using Eq. 2.

*Material*	*Energy range (keV)*	*a_1_*	*a_2_*	*a_3_*	*a_4_*	*a_5_*	*Absolute maximum percentage difference*
Hydrogen	1-5	1.017E-2	−1.267E-4	4.941E-6	6.211E-7	−4.419-10	< 1.0
	5-20	2.326E-2	−1.389E-3	1.539E-4	−1.327E-5	2.106E-7	< 1.0
	20-50	6.026E-2	−3.383E-2	2.032E-2	−6.446E-3	4.520E-4	2.4
	50-400	6.204E-2	−3.152E-2	−3.056E-3	1.944E-2	−6.442E-3	~0.5
Carbon	1-8	1.302	−6.782E-2	6.730E-3	1.085E-4	−4.601E-7	1.7
	8-50	2.445E-2	−6.282E-3	2.236E-3	4.060E-4	−3.192E-6	2.0
	50-400	3.163E-2	−2.176E-2	1.655E-2	−5.462E-3	5.521E-4	~ 0.5
Nitrogen	1-8	2.531	−1.265E-1	1.243E-2	9.415E-5	−5.600E-7	2.0
	8-50	2.518E-2	−7.491E-3	3.281E-3	7.909E-4	−1.036E-5	1.0
	50-400	3.155E-2	−2.077E-2	1.395E-2	−2.583E-3	9.600E-5	~ 0.3
Oxygen	1-8	3.049	−1.711E-1	1.903E-2	9.178E-5	−8.530E-7	1.2
	8-50	2.773E-2	−1.051E-2	5.376E-3	1.212E-3	−1.808E-5	< 1.0
	50-400	3.137E-2	−1.928E-2	1.113E-2	6.494E-4	−3.798E-4	~ 0.5
Florine	1-5	6.132	−2.645E-1	2.795E-2	−9.100E-6	−9.025E-7	~ 0.1
	5-30	5.527E-2	−2.417E-2	1.067E-2	1.208E-3	−1.515E-5	~ 1.0
	30-80	1.526E-2	2.054E-2	−2.862E-2	1.822E-2	−1.796E-3	1.7
	80-400	2.974E-2	−1.833E-2	1.091E-2	1.926E-3	−3.648E-4	~ 0.4
Sodium	2-5	−5.450E+1	1.126E+0	−1.249E-2	2.455E-3	−1.623E-5	< 1.0
	5-20	1.927E-1	−6.497E-2	2.586E-2	1.646E-3	−2.470E-5	< 1.0
	20-100	2.907E-2	−1.791E-2	1.512E-2	2.991E-3	−2.668E-5	< 1.0
	100-400	3.002E-2	−1.876E-2	1.329E-2	5.421E-3	−6.481E-4	~ 1.0
Magnesium	2-8	1.169E+0	−2.210E-1	5.431E-2	2.878E-4	−7.704E-6	< 1.0
	8-20	7.318EE-2	−5.666E-2	3.207E-2	2.462E-3	−4.147E-5	< 1.0
	20-100	2.924E-2	−1.719E-2	1.624E-2	5.920E-3	−1.992E-4	< 1.0
	100-400	3.130E-2	−2.230E-2	2.133E-2	3.853E-3	−3.205E-5	0.1
Aluminum	2-8	3.058	−3.594E-1	7.575E-2	−3.440E-4	−5.329E-6	~ 0.1
	8-50	8.252E-2	−6.097E-2	3.856E-2	3.343E-3	−6.740E-5	~ 1.6
	50-400	3.027E-2	−2.150E-2	1.843E-2	9.644E-3	−6.815E-4	~ 0.5
Silicon	2-10	5.080	−5.491E-1	1.085E-1	−1.478E-3	6.659E-7	~ 1.3
	10-50	4.625E-2	−4.078E-2	3.813E-2	6.765E-3	−1.908E-4	~ 0.2
	50-400	3.135E-2	−2.004E-2	1.902E-2	6.646E-2	−1.652E-3	~ 0.4
Phosphorus	3-10	−6.277E+0	2.293E-1	5.475E-2	3.295E-3	−5.545E-5	< 1.0
	10-50	3.917E-2	−4.013E-2	4.427E-2	8.548E-3	−2.700E-4	< 1.0
	50-400	3.051E-2	−2.037E-2	2.288E-2	1.957E-2	−1.873E-3	< 1.0
Sulphur	3-10	−3.393E+1	2.155	−9.218E-2	1.521E-2	−1.895E-4	1.4
	10-50	5.276E-2	−5.911E-2	6.259E-2	9.104E-4	−2.969E-4	< 1.0
	50-200	3.286E-2	−3.143E-2	4.686E-2	1.237E-2	−1.849E-4	< 0.2
	200-400	3.062E-2	−1.328E-2	1.102E-2	3.532E-2	−3.218E-3	~ 0.8
Chlorine	3-5	2.508e+2	2.335	−6.149E-1	6.954E-2	−9.041E-4	< 0.1
	5-20	3.713E-1	−2.064E-1	1.181E-1	2.063E-3	−8.765E-5	< 0.1
	20-100	3.276E-2	−3.378E-2	5.209E-2	1.707E-2	−8.244E-4	< 0.2
	100-400	2.937E-2	−1.193E-2	1.290E-2	4.088E-2	−3.706E-3	~ 1.0
Argon	4-20	1.288	−4.119E-1	1.679E-1	−2.568E-3	−1.559E-5	< 0.5
	20-80	5.042E-2	−6.961E-2	8.440E-2	1.078E-2	−4.203E-4	< 1.0
	80-400	2.860E-2	−2.150E-2	3.728E-2	3.205E-2	−2.929E-3	~ 0.5
Potassium	4-20	6.425E-1	−3.714E-1	1.985E-1	−3.001E-3	−2.719E-3	0.3
	20-100	3.789E-2	−4.925E-2	8.218E-2	2.211E-2	−1.202E-3	0.1
	100-400	3.090E-2	−2.313E-2	4.542E-2	4.153E-2	−3.093E-3	~ 0.6
Calcium	5-20	1.370E+0	−5.903E-1	2.700E-1	−8.590E-3	4.930E-5	< 1.0
	20-100	3.938E-2	−5.474E-2	9.881E-2	2.675E-2	−1.588E-3	0.1
	100-400	3.169E-2	−2.455E-2	5.544E-2	4.973E-2	−3.767E-3	~ 0.4
Manganese	8-30	3.641E-1	−4.043E-1	3.777E-1	−1.380E-2	−1.170E-4	~ 0.1
	30-60	1.047E-1	−2.588E-1	3.858E-1	−4.620E-2	5.414E-3	~ 0.1
	60-400	2.917E-2	−2.751E-2	1.049E-1	9.505E-2	−1.227E-2	~ 0.6
Iron	8-40	4.584E-1	−4.655E-1	4.374E-1	−1.884E-2	−1.466E-4	0.1
	40-100	5.345E-2	−1.206E-1	2.574E-1	3.069E-2	−3.080E-3	0.1
	100-400	2.983E-2	−2.947E-2	1.248E-1	1.069E-1	−1.430E-2	0.2

**Table 3:** Elemental compositions and mass density of selected body tissues

*Material*	*H*	*C*	*N*	*O*	*F (fraction by weight)*	*Ca*	*Other*	*Density (g. cm^−3^)*
From Table 2 of reference 15 of this text								
A-150 tissue-Equivalent plastic	0.101330	0.775498	0.035057	0.052315	0.017423	0.018377	-	1.127
Bone, cortical	0.034000	0.155000	0.042000	0.435000	-	0.225000	0.001 Na, 0.002 Mg, 0.103 P, 0.003 S	1.920
Breast tissue	0.106000	0.332000	0.030000	0.527000	-	-	0.001 Na, 0.001 P, 0.002 S, 0.001 Cl	1.020
Lung tissue	0.103000	0.105000	0.031000	0.749000	-	-	0.002 Na, 0.001 P, 0.003 S, 0.003 Cl, 0.002 K	1.050
Muscle, skeletal	0.102000	0.143000	0.034000	0.710000	-	-	0.001 Na, 0.001 P, 0.002 S, 0.001 Cl, 0.004 K	1.050
Polymethyl	0.080541	0.599846	-	0.319613	-	-	-	1.190
Methacrylate								
From Appendix A of ICRU 44 (reference 37 of this text): Adipose tissue:								
Newborn #2	0.111000	0.297000	0.009000	0.580000	-	-	0.001 Na, 0.001 S, 0.001 Cl	0.990
Infant (2 days-10 months) #2	0.112000	0.392000	0.009000	0.484000	-	-	0.001 Na, 0.001 S, 0.001 Cl	0.970
Child (1-18 years) #2	0.113000	0.445000	0.006000	0.433000		-	0.001 Na, 0.001 S, 0.001 Cl	0.960
Adult #2	0.116000	0.681000	0.002000	0.198000	-	-	0.001 Na, 0.001 S, 0.001 Cl	0.990

## Discussion

The maximum percentage differences between the values of mass attenuation and mass energy absorption coefficients obtained from Eq. (1) and those fitted are about 2.0% over a large range of energy of photons [Tables 1 and 2]. Figures 1 and 2 show that the values of mass attenuation and mass-energy absorption coefficients obtained from Eqs. 1–3 and those reported by Hubbell and Seltzer[15] are in good agreement. However, the values of mass attenuation and mass-energy absorption coefficients differ as a result of difference in age [Figure 3]. This difference is attributable to the variations in fraction-by-mass of elements in the body tissue for different ages [Table 3]. For instance, for adipose tissue, the values of mass attenuation and mass-energy absorption coefficients for newborns (in the energy range between 1 keV and 100 keV) differ by 8.0 and 9.0% respectively when compared with those for infants. These differences are 12.0 and 13.0% respectively when comparisons are made between newborns and children. For adults in comparison with newborns, these differences are 25.0 and 27.0% respectively [Figure 3]. Figure 3 shows that the variations of the values of mass attenuation and mass energy-absorption coefficients are remarkably high (maximum of up to 50%) for skeleton-cortical bone for different ages in the energy of photons ranging between 1 keV and 100 keV.

The parameterization and the computer program developed in this work for the evaluation of mass attenuation and mass energy-absorption coefficients are of tremendous usefulness in diagnostic and therapeutic medical procedures. Firstly, the development of ‘body tissue’-equivalent materials requires the matching of the attenuation and absorption characteristics with those of ideal tissue. Secondly, the absorbed doses in biological medium (or body tissue) and dosimeter are related by the ratios of the mass energy-absorption coefficients of X-rays in these media. It is desirable to have tissue-equivalent materials formulated in such a way as to have the same/close elemental composition as the ideal. Producing an exact matching of body tissue and tissue substitutes with the same elemental composition seems practically unachievable. However, both tissue substitute and ideal body tissue are considered to be equivalent if they exhibit the same or close attenuation and absorption properties. Theoretically, this equivalence can be simulated by having the values of (μ/ρ)_substitute_/(μ/ρ)_tissue_ and (μ_en_/ρ)_substitute_/(μ_en_/ρ)_tissue_ equal to unity across a wide range of energy distribution of photons. In order to assist interested parties in developing substitutes for body tissues, tables of elemental composition by percentage weight, (μ/ρ)_substitute_/(μ/ρ)_tissue_, (μ_en_/ρ)_substitute_/(μ_en_/ρ)_tissue_ and densities for some body tissues and 64 tissue substitutes over 33 energy points ranging between 0.01 and 100 MeV have been reported.[38]

The exact knowledge of the elements in an ideal body tissue and their percentage by weight is crucial for achieving optimum equivalence in the simulation of substitutes for body tissues. It is not a trivial phenomenon to know the exact percentage by weight of elements of body tissues. The percentage by weight of elements of body tissues varies with age, gender and state of health (International Commission on Radiation Units and Measurements, ICRU Report 46, 1992).[39] The issue of the determination of the elemental compositions of body tissues and the percentage by weight of constituent elements has been addressed by several authors.[18,40,41] Largely, body tissues are known to be made up of oxygen, carbon, hydrogen, nitrogen, calcium, phosphorus, sulfur, potassium, sodium, chlorine, magnesium and iron. A recent review of the experimental methods for the evaluation of atomic, molecular and cellular composition of body tissues/organs has been published.[42] As a result of the differences in tissue samples and experimental techniques reported in widely consulted literatures, there are significant disparities in values of percentage of some elements reported by workers for the same tissue. In the report published by the ICRU,[39] it was noted that *‘… it is imperative that body tissue compositions are not given the standing of physical constants and their expected variability is always taken into consideration…’* Consequently, due to these uncertainties, this publication[39] reported sets of radiation-interaction data to illustrate the spread of elemental compositions for different ages, genders and states of health. Figure 3 shows that there are significant variations in the values of mass attenuation and mass energy-absorption coefficients for the same tissue for individuals with different ages. Uncertainties in the composition of body tissue and radiation-interaction coefficients are sources of uncertainties or errors in the estimation of absorbed dose.[39] It is not practically possible to have all radiation-interaction data tabulated for different varieties of tissues for different ages, genders and states of health. The use of less precise values of mass attenuation and mass energy-absorption coefficients or failure to apply appropriate correction factors could result in significant errors in the simulation of body tissues.

## Conclusion

The parameterization and computer programs, MUA_T and MUEN_T, that are reported in this work provide for the evaluation of mass attenuation and mass energy-absorption coefficients for a given body tissue or substitute of arbitrary percentage-by-weight elemental composition. These can serve as technical tools in the optimization studies involving the formulation of phantoms for body tissues in low-energy diagnostic radiology and orthovoltage therapeutic applications. In terms of the optimization of speed and memory utilization, it is preferable to use mathematical expression rather than interpolation to obtain interaction data at desired intermediate energies that are not tabulated in literatures. Among the various interpolation techniques used in obtaining $\frac{\text{μ}}{\text{ρ}}\left(x\right)$ and $\frac{{\text{μ}}_{\text{en}}}{\text{ρ}}\left(x\right)$ data, the log-log cubic spline interpolation method is considered to produce more accurate results. However, this technique requires more computer memory storage and run time when compared with the use of functional expression. The functional expressions reported in this work can provide opportunity for reduction in data storage requirements and computation time, most especially in extensive computer programs requiring the use of$\frac{\text{μ}}{\text{ρ}}\left(x\right)$ and $\frac{{\text{μ}}_{\text{en}}}{\text{ρ}}\left(x\right)$ data, and the log-log cubic data for compounds and mixtures.
